# Profile and correlates of functional status in elderly patients presenting at a primary care clinic in Nigeria

**DOI:** 10.4102/phcfm.v7i1.810

**Published:** 2015-07-13

**Authors:** Samuel A. Ajayi, Lawrence A. Adebusoye, Adetola M. Ogunbode, Joshua O. Akinyemi, Ayodeji M. Adebayo

**Affiliations:** 1 Family Medicine Department, University College Hospital, Ibadan, Nigeria; 2 Department of Epidemiology and Medical Statistics, College of Medicine, University of Ibadan, Nigeria; 3 Department of Preventive Medicine and Primary Care, College of Medicine, University of Ibadan, Nigeria

## Abstract

**Background:**

Assessing the functional status of elderly patients is central in measuring their health outcome. Little is known about the functional status of elderly patients attending our primary care clinic in Nigeria.

**Objective:**

To assess the correlates of functional status in elderly patients presenting at the General Outpatient Clinic of the University College Hospital, Ibadan, Nigeria.

**Method:**

A cross-sectional study of 360 randomly selected patients aged 60 years and above was undertaken to assess their functional status by scoring their basic activities of daily living (BADL) using the Modified Bathel Index. An interviewer-administered questionnaire was used to obtain the socio-demographic data, anthropometric measurements and morbidities of each patient.

**Results:**

The mean age was 69.1 ± 6.6 years with a female-to-male ratio of 1.9: 1. The prevalence of overall functional disability (defined as when assistance was sought in the performance of at least one of the components of BADL) was 88.3%. The highest prevalence of functional disability was experienced in the area of personal hygiene and grooming (95.3%) and transferring from bed to chair (95.3%). Overall functional disability significantly increased with increasing age (χ^2^ for trend=14.004, *p* < 0.0001), living in a polygamous family unit (*p* = 0.025), and lack of formal education (*p* = 0.020).

**Conclusion:**

Functional disability was high amongst the elderly in this setting. Age, education, and living in a polygamous type of family unit had significant influence on the functional status. High premium should, therefore, be placed on considering these factors in reducing functional disability in the elderly.

## Abstract (fr)

**Profil et correspondances du statut fonctionnel des personnes âgées qui se présentent àune clinique de soins primaires au Nigéria.**

**Contexte:**

L’é valuation de la situation fonctionnelle des personnes âgées est essentielle pour mesurer leurs résultats en matière de santé. On ne sait pas grand-chose sur la situation fonctionnelle des patients âgés qui fréquentent notre clinique de soins primaires au Nigéria.

**Objectif:**

Evaluer les correspondances du statut fonctionnel des patients âgés qui fréquentent la policlinique générale de l’Hôpital académique (UCH), Ibadan, Nigeria.

**Méthode:**

Une étude transversale de 360 patients sélectionnés au hasard, âgés de 60 ans et plus, a été faite de septembre ànovembre 2011. On a évalué le statut fonctionnel en mesurant leurs activités courantes de leur vie quotidienne (ACVQ) au moyen de l’Indice Bathel Modifié (MBI). On a utilisé un questionnaire administré par un interviewer pour obtenir les données sociodémographiques, les mesures anthropométriques et les morbidités de chaque patient.

**Résultats:**

L’â ge moyen était de 69.1 ± 6.6 ans avec une proportion femme-homme de 1.9: 1.La prédominance d’incapacité fonctionnelle générale (définie comme le moment où on a besoin d’aide pour effectuer au moins l’une des composantes d’ACVQ) était de 88.3%. La prédominance d’incapacité fonctionnelle se ressentait dans le domaine de l’hygiène personnelle et de la toilette (95.3%) et dans le transfert du lit àla chaise (95.3%). L’incapacité fonctionnelle générale augmentait considérablement avec l’â ge (χ^2^ pour la tendance = 14.004, *p* < 0.0001), la vie dans une famille de type polygame (*p* = 0.025) et le manque d’é ducation formelle (*p* = 0.020). Dans cette étude les morbidités détectées n’é taient pas vraiment liées au statut fonctionnel.

**Conclusion:**

L’incapacité fonctionnelle était élevée chez les personnes âgées dans ce contexte. Les facteurs sociodémographiques, particulièrement l’â ge, l’é ducation, et la vie dans un type de famille polygame avaient plus d’influence sur le statut fonctionnel que les comorbidités. Il faudra donc mettre plus l’accent sur ces facteurs pour diminuer l’incapacité fonctionnelle chez les personnes âgées.

## Introduction

The global demographic transition has caused an unprecedented increase in the elderly population with a higher rate of increase in the developing countries like Nigeria.^1,2^ The United Nations General Assembly defines an elderly person as a person aged 60 years and above, whilst the World Health Organization (WHO) puts the age cut-off at 65 years and above.^3^ Most developing countries use 60 years and above due to lower life expectancies and proportions of older persons within the population.

Population ageing is associated with increased morbidity and demand on healthcare services.^4^

Multi-morbidities, illness chronicity, non-specific presentations, and deranged social factors characterise the health profiles of the elderly. Therefore, assessment of functional status is critical as it provides more holistic evaluation and care for these older patients

Since 1959, the WHO asserted that health in the elderly is best measured in terms of function.^5^ This is a better indicator of health in the elderly than the traditional diagnostic categories. Traditionally, health and the outcomes of treatment have been measured with regard to morbidity, mortality, incidence, or prevalence of disease. For chronic diseases, however, and particularly in the elderly, functional health status as measured by Activities of Daily Living (ADL) is now an important measure, especially in primary care.^6^ Functional status is defined as the individual ability to perform normal daily activities required to meet basic needs, fulfil usual roles and maintain health and well-being.^7^ ADL is used in rehabilitation as an umbrella term relating to self-care, and comprises those activities or tasks that people undertake routinely in their everyday lives. Basic activities of daily living (BADL), which is often used synonymously as ADL, refer to the basic tasks of everyday life, such as eating, bathing, dressing, toileting, and transferring.

Geriatrics as a medical specialty is yet to fully emerge in Nigeria. For this reason there is a paucity of information on the morbidity pattern, functional status and its determinants in the elderly.8,9 Most of the previous studies were carried out in developed populations, but these may not be applicable in a developing country like Nigeria. A few available local studies were community-based, however, this hospital-based study provides baseline data that will be useful for effective planning in a primary care setting with a growing elderly population.

## Ethical consideration

Ethical clearance for the study was obtained from the joint University of Ibadan and University College Hospital Ethical Review Board and the Head of the Department. The respondents either signed or thumb-printed the informed consent form before being studied. They were assured of confidentiality. The questionnaires were serially coded and the data entered on the computer. The data were protected and accessible only to the researcher, data entry clerk and statistician.

## Method

### Study Setting and participants

This study was conducted at the General Outpatients (GOP) Clinic, Family Medicine Department, University College Hospital (UCH), Ibadan. Ibadan is the capital city of Oyo State, southwest Nigeria, with an estimated population of 2.55 million according to the 2006 National Population Census. UCH is a 1000-bed tertiary academic institution that was founded in 1957. Patients from across Nigeria and the west-African subregion are referred to the hospital. The General Outpatients Clinic serves as a primary care clinic within a tertiary hospital setting, as most patients seen at UCH are managed at first contact, and few are subsequently referred to specialty units and other medical services like physiotherapy and clinical psychology. Elderly patients constitute about 16% of the 1200 new adult patients seen every month in this clinic.

The study population comprised 360 elderly male and female patients aged 60 years and above attending the GOPD clinic, UCH, between 08 September and 30 November 2011. The age of the respondents was determined by direct recall, use of historical events, marital age, and age of their first child.

### Study design

This was a cross-sectional descriptive study which assessed the functional status of 360 elderly respondents. A simple random sampling technique was used by means of a computer-generated table of random numbers to select a sample of 360 elderly persons 60 years and over presenting at the GOP clinic. To recruit the total sample size of 360 respondents over 60 working days, six respondents who met the inclusion criteria were selected daily. From the Health Record Section of the GOP, a total of 1187 elderly patients were seen both as newly-presenting and follow-up patients over the same study period in the previous year, from September to November 2010. This gave an average of 20 elderly patients seen per working day. Random numbers were then generated using the Microsoft Excel package of the Windows XP programme, six randomly selected numbers were generated daily. These were the numbers of the patients recruited daily. All non-consenting elderly and critically ill patients were excluded from the study.

### Procedure

**FIGURE 1:**
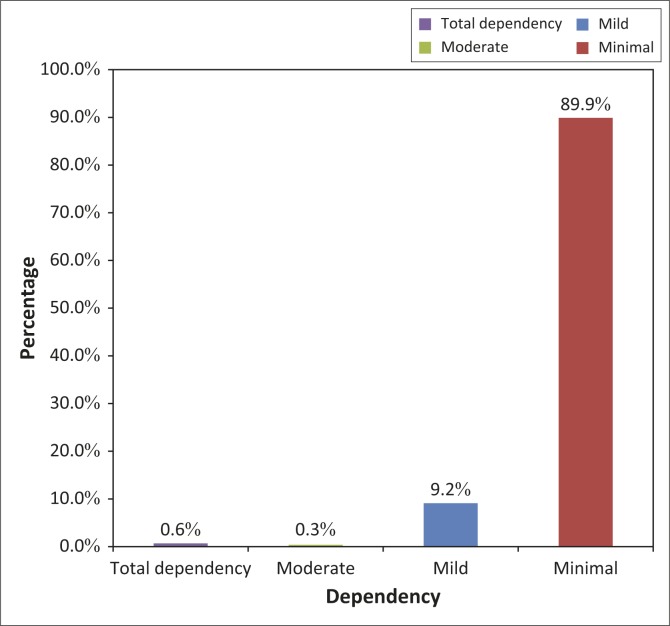
Level of Dependency amongst the respondents with functional disability.

The study involved administration of a structured interviewer-administered questionnaire and a general medical examination. The functional assessment was carried out by scoring the respondents’ BADL using the Modified Barthel Index (MBI) incorporated into the questionnaire. The maximum obtainable score was 100. The higher the score, the more ‘independent’ the individual. The score for each patient was used to predict his or her dependency needs.^10,11^ Scores between 0–24 were classified as total dependency; 25–49 as severe dependency; 50–74 as moderate dependency; 75–90 and 91–99 as mild and minimal dependency respectively according to the MBI instrument. For the purpose of comparison of the results of this study with other studies, BADL performance scores were dichotomised into functional independence and functional disability. In this study, functional independence was defined as ability to fully perform all the components of the BADL using MBI without any assistance, whilst functional disability was defined as when assistance was sought in the performance of at least one of the components of BADL^12,13,14^(see Figure 1).

Thereafter, the respondents were examined, and urine chemistry and packed cell volumes were measured. Some of the respondents were referred to other specialty units within UCH for further assessment and management.

### Statistical analysis

Administered questionnaires were checked, sorted and coded serially after each study day. SSPS (version 17) was used for data entering, cleaning and analysis. Descriptive statistics were used to describe the socio-demographic characteristics of the respondents. The chi-square test was used to evaluate the association between categorical variables at 5% level of significance.

## Results

The mean age was 69.1 ± 6.6 years. The majority of the respondents (65%) were females, with a female-to-male ratio of 1.9:1. The modal age group was 70–74 years for the men and 60–64years for the women.

Only 42 (11.7%) of the respondents were functionally independent, whilst the rest had functional disability. Amongst the latter, the majority (89.9%) were minimally dependent, with only two respondents (0.6%) having total dependency (see Table 1).

**TABLE 1:** Socio-demographic characteristics of the respondents.

Socio-demographic Characteristics	Male = 126 *n* (%)	Female = 234 *n* (%)	*N* = 360 Total (%)
**Age group (years)**			
60–64	29 (23.0)	72 (30.8)	101 (28.1)
65–69	28 (22.2)	65 (27.8)	93 (25.8)
70–74	36 (28.6)	46 (19.7)	82 (22.8)
75–79	18 (14.3)	31 (13.2)	49 (13.6)
80 and above	15 (11.9)	20 (8.5)	35 (9.7)
**Marital status**			
Married	116 (92.1)	100 (42.8)	216 (60.0)
Separated/Divorced	7 (5.5)	12 (5.1)	19 (5.3)
Widowed	3 (2.4)	122 (52.1)	125 (34.7)
**Family type**			
Monogamous	86 (68.3)	87 (37.3)	173 (48.1)
Polygamous	40 (31.7)	147 (62.7)	187 (51.9)
**No. of children**			
< 5 children	27 (21.4)	94 (40.2)	121 (33.6)
≥ 5 children	99 (78.6)	140 (59.8)	239 (66.4)
Ethnicity			
Yoruba	119 (94.4)	225 (96.2)	344 (95.6)
Ibo	1 (0.8)	4 (1.7)	5 (1.4)
Others**	6 (4.8)	5 (2.1)	11 (3.0)
**Religion**			
Christianity	70 (55.5)	130 (55.6)	200 (55.6)
Islam	55 (43.7)	103 (44.0)	158 (43.8)
Others	1 (0.8)	1 (0.4)	2 (0.6)
Education			
Number formal education	39 (31.0)	133 (56.8)	172 (47.8)
Primary education	38 (30.1)	60 (25.7)	98 (27.2)
Secondary education	15 (11.9)	22 (9.4)	37 (10.3)
Tertiary education	34 (27.0)	19 (8.1)	53 (14.7)
**Occupational status**			
Currently engaged	85 (67.5)	208 (88.9)	293 (81.4)
Currently unengaged	41 (32.5)	26 (11.1)	67 (18.6)
Income			
< $1.25/day	53 (42.1)	174 (74.4)	227 (63.1)
≥ $1.25/day	73 (57.9)	60 (25.6)	133 (36.9)

**TABLE 2:** Functional status in each components of BADL by gender.

Functional status	Male = 126 *n* (%)	Female = 234 *n* (%)	*N* = 360 Total (%)	χ^2^, *p*-value
**Personal hygiene/grooming**				
Functionally independent	8 (6.3)	9 (3.8)	17 (4.7)	χ^2^ = 1.140,
Functional disability	118 (93.7)	225 (96.2)	343 (95.3)	*p* = 0.286
**Bathing self**				
Functionally independent	110 (87.3)	193 (82.5)	303 (84.2)	χ^2^ = 1.430,
Functional disability	16 (12.7)	41 (17.5)	57 (15.8)	*p* = 0.232
**Feeding**				
Functionally independent	109 (86.5)	157 (67.1)	266 (73.9)	χ^2^ = 15.999,
Functional disability	17 (13.5)	77 (32.9)	94 (26.1)	*p* < 0.0001*
**Toilet use**				
Functionally independent	12 (9.5)	15 (6.4)	27 (7.5)	χ^2^ = 1.144,
Functional disability	114 (90.5)	219 (93.6)	333 (92.5)	*p* = 0.285
**Stair climbing**				
Functionally independent	17 (13.5)	19 (8.1)	36 (10.0)	χ^2^ = 2.627,
Functional disability	109 (86.5)	215 (91.9)	324 (90.0)	*p* = 1.105
**Dressing**				
Functionally independent	21 (16.7)	30 (12.8)	51 (14.2)	χ^2^ = 0.996,
Functional disability	105 (83.3)	204 (87.2)	309 (85.8)	*p* = 0.316
**Bowel control**				
Functionally independent	8 (6.3)	16 (6.8)	24 (6.7)	χ^2^ = 0.031,
Functional disability	118 (93.7)	218 (93.2)	336 (93.3)	p = 0.859
**Bladder control**				
Functional independent	10 (7.9)	18 (7.7)	28 (7.8)	χ^2^ = 0.007,
Functional disability	116 (92.1)	216 (92.3)	332 (92.2)	p = 0.934
**Mobility**				
Functionally independent	12 (9.5)	19 (8.1)	31 (8.6)	χ^2^ = 0.205,
Functional disability	114 (90.5)	215 (91.9)	329 (91.4)	*p* = 0.651
**Chair/bed transfer**				
Functionally independent	7 (5.6)	10 (4.3)	17 (4.7)	χ^2^ = 0.299,
Functional disability	119 (94.4)	224 (95.7)	343 (95.3)	*p* = 0.584

BADL, basic activities of daily living.

*, Significant at 5% level of significance

The functional status of the respondents was further assessed based on the components of BADL by gender (Table 2). Overall, functional disability was most prevalent in the performance of personal hygiene, grooming and transferring from the bed to the chair, with 95.3% of the respondents having functional disability in each activity. The men were found to have more disability with bowel control than the women, although this was not statistically significant.

**TABLE 3:** Association between socio-demographic factors and respondents’ functional status.

Variable	Functional Disability (%) *n* = 318	Functionally Independent (%) *n* = 42	Total (%) (*n* = 360)	χ^2^, *p*-value
**Age (Years)**				
60-64	79 (78.2)	22 (21.8)	101 (100.0)	**χ**^2^ = 14.004
65-69	84 (90.3)	9 (9.7)	93 (100.0)	*p* < 0.0001*
70-74	74 (90.2)	8 (9.8)	82 (100.0)	
75-79	46 (93.9)	3 (6.1)	49 (100.0)	
≥ 80	35 (100.0)	0 (0.0)	35 (100.0)	
**Gender**				
Male	114 (90.5)	12 (9.5)	126 (100.0)	**χ**^2^= 0.864
Female	204 (87.2)	30 (12.8)	234 (100.0)	*p* = 0.353
**Marital Status**				
Currently Married	192 (88.9)	24 (11.1)	216 (100.0)	χ^2^= 0.162
Not married	126 (87.5)	18 (12.5)	144 (100.0)	*p* = 0.688
**Family type**				
Monogamous	146 (84.4)	27 (15.6)	173 (100.0)	χ^2^= 5.018
Polygamous	172 (92.0)	15 (8.0)	187 (100.0)	*p* = 0.025*
**No of children**				
< 5 children	102 (84.3)	19 (15.7)	121 (100.0)	**χ**^2^= 2.881
≥ 5 children	216 (90.4)	23 (9.6)	239 (100.0)	*p* = 0.090
**Education**				
No formal Education	159 (92.4)	13 (7.6)	172 (100.0)	**χ**^2^= 5.395
Formal Education	159(84.6)	29 (15.4)	188(100.0)	*p* = 0.020*
**Occupation**				
Currently unengaged	60 (89.6)	7 (10.4)	67 (100.0)	**χ**^2^= 0.119
Currently engaged	258 (88.1)	35 (11.9)	293 (100.0)	*p* = 0.730
**Income**				
Below poverty level	204 (89.9)	23 (10.1)	227 (100.0)	**χ**^2^= 1.404
Above poverty level	114 (85.7)	19 (14.3)	133 (100.0)	*p* = 0.236

*, Significant at 5% level of significance

The association between socio-demographic characteristics and respondents’ functional status is shown in Table 3. Extended Mantel-Haensel chi-square for trend showed that functional disability increased significantly with increasing age from 78.2% in the age group 60–64 years, to 100.0% at age 80 years and above (*p* < 0.0001). Significantly, a higher proportion of respondents in polygamous family settings (92.0%) had functional disability compared with those in the monogamous families (84.4%) (*p* = 0.025). The prevalence of functional disability (84.6%) was less amongst respondents with some formal education compared to those without formal education (92.4%) (*p* = 0.020).

Table 4 depicts the association between the commonest morbidities diagnosed and the functional status of the respondents. Functional disability was higher in those visually impaired with cataracts and glaucoma, osteoarthritis, diabetes mellitus, urinary tract infection and men with erectile dysfunction. There was no significant association between co-morbidities and functional status.

**TABLE 4:** Association between common morbidities and respondents’ functional status.

Common morbidities	Functional disability (%) *n* = 318	Functionally Independent (%) *n* = 42	Total (%) (*n* = 360)	*χ*^2^, *p*-value
**Hypertension**				
Yes	211 (86.5)	33 (13.5)	244 (100.0)	χ^2^= 2.536,
No	107 (92.2)	9 (7.8)	116 (100.0)	*p* = 0.111
Cataract				
Yes	83 (93.3)	6 (6.7)	89 (100.0)	χ^2^= 2.783,
No	235 (86.7)	36 (13.3)	271 (100.0)	*p* = 0.095
**Osteoarthritis**				
Yes	98 (89.9)	11 (10.1)	109 (100.0)	χ^2^= 0.376,
No	220 (87.6)	31 (12.4)	251 (100.0)	*p* = 0.540
Diabetes Mellitus				
Yes	48 (94.1)	3 (5.9)	51 (100.0)	χ^2^ = 1.929,
No	270 (87.4)	39 (12.6)	309 (100.0)	*p* = 0.165
**Glaucoma**				
Yes	17 (89.5)	2 (10.5)	19 (100.0)	*p* = 1.000^+^
No	301 (88.3)	40 (11.7)	341 (100.0)	
**Malaria**				
Yes	13 (86.7)	2 (13.3)	15 (100.0)	*p* = 0.690*
No	305 (88.4)	40 (11.6)	345 (100.0)	
**Urinary Tract Infection**				
Yes	9 (90.0)	1 (10.0)	10 (100.0)	*p* = 1.000*
No	309 (88.3)	41 (11.7)	350 (100.0)	
**Refractive error**				
Yes	6 (66.7)	3 (33.3)	9 (100.0)	*p* = 0.075*
No	312 (88.9)	39 (11.1)	351 (100.0)	
**Respiratory Tract Infection**				
Yes	8 (88.9)	1 (11.1)	51 (100.0)	*p* = 1.000*
No	310 (88.3)	41 (11.7)	309 (100.0)	
**Erectile Dysfunction**†	*n* = 114	*n* = 12	*n* = 126	*p* = 0.070*
Yes	24 (100.0)	0 (0.0)	24 (100.0)	
No	90 (88.2)	12(11.8)	102 (100.0)	

†, Male only, + Fishers Exact Test.

*, Significant at 5% level of significance

## Discussion

The profile and correlates of functional status are critical in ensuring holistic care for the elderly. These measurements have been found to be better indicators of health in the elderly than the traditional diagnostic categories. In this study, estimates of functional disability amongst elderly attendees at a primary care clinic were reported. Information on the socio-demographic and morbidity correlates was also provided.

The prevalence of functional disability in this study was very high (88.3%). Functional disability prevalence varies worldwide due to differences in definition criteria, measurement, and sample characteristics.^15^ The prevalence is expected to be high in studies in which functional disability is based on any level of assistance in the performance of daily activities as it applied in this study.^9^ Such high prevalence as found in this hospital-based study could also be expected if performed in an institutionalised setting such as an old persons’ nursing home. This prevalence was much higher than what was reported in a study using the same instrument in Northern Nigeria (28.3%).^13^ The reason for a higher prevalence in our study might be because it was hospital-based, whilst the northern Nigerian study was community-based. The cultural perception of the performance of BADL could also influence the respondents’ scores. Requesting for assistance is a common and acceptable practice amongst the elderly in African culture, irrespective of his or her ability to carry out an activity or task. This high level of functional disability does not translate to high level of physical handicap, as was corroborated by the fact that well over three-quarters of the respondents were minimally dependent in carrying out the basic activities of daily living (BADL), which translated to needing less than ten hours of help per week.^10^

Given the definition, study setting, and cultural caveats, the prevalence of functional disability was much higher than reported in a few similar community-based studies done previously in this environment, Europe and Asia.^9,13,16,17,18^ For performance in the individual components of BADL however, the women were found to have an increased level of disability, except for bowel control. The higher prevalence of physical disability for the BADL components amongst elderly women compared to elderly men in this study have been reported in previous studies.^19,20^ Whilst women, globally, live longer, functional disabilities are more prevalent amongst them.^21,22^ The cumulative effect of pregnancy and childbearing, lower level of education, and poor health care may be responsible for these higher physical dependencies and functional limitations seen in elderly women. This study also showed that the majority of the respondents were still able to carry out activities that are largely dependent on the strength of the upper extremities, such as feeding and bathing, without any assistance. In the loss of independence in BADL activities, studies have shown that this is the last set of activities to be lost.^5,23^

As with most of the previous reports, advanced age and polygamy were significantly associated with functional disability in the performance of BADL, with the oldest of the old clearly having the poorest functional status.^9,13,24^ All the respondents (100%) above 80 years had functional disability, which was comparable with a prevalence of 98.9% found in a community-based study in Northern Nigeria, but well above 50% recorded by the elderly Europeans.^13^ Availability of better health delivery and assisting devices in the performance of BADL may be responsible for lower prevalence amongst the Europeans. The increasing trend of functional disability with increasing age has, however, been reported in all previous studies and corroborated by the WHO.^9,13,25^ This, Chappell and Cooke opined, might be due to the increasing prevalence of chronic illnesses as ageing sets in.^12^

Expectedly, this study further revealed that functional disability was more prevalent amongst respondents living below poverty level and those who lack formal education. The same trend has been observed in the studies reviewed.^15,25^ Poverty, ignorance, diseases and consequent disability is a vicious cycle, which Mont asserted are inseparable.^15^ Poverty leads to malnutrition, poor health services and sanitation, as well as unsafe living and working conditions, which are all associated with disability.

None of the common morbidities were significantly related to functional status. The failure to find significant elevated risk of functional disability amongst those with a medical condition was also reported in the Ibadan Study of Ageing.^9^This may reflect a reporting bias: poor access to medical service as well as a high level of illiteracy, which would limit the number of elderly persons who might be aware of having a medical condition, especially asymptomatic diseases such as hypertension.^9,26^ Some of the morbidities were associated with a higher prevalence of functional disability, although this was not statistically significant. These include cataract, osteoarthritis, diabetes mellitus, glaucoma, UTI and RTI. Cataract and glaucoma are the major causes of visual impairment, which will obviously affect the ability to carry out BADL. Osteoarthritis reduces joint mobility and inflicts pain, which may explain why its occurrence in this study was associated with a higher prevalence of functional disability.

## Limitations of the study

This was a cross-sectional study. As such, causal relationships between functional status and the variables implicated cannot be strongly drawn. It was a hospital-based study, therefore the likely generalisability of the findings is limited.

## Conclusion and recommendations

The prevalence of functional disability in the overall performance of BADL was very high amongst the study population, whilst increasing age, polygamy, illiteracy, and poverty were associated factors. These factors had more influence than co-morbidities on functional status. Therefore, a high premium should be placed on considering these factors in caring for the elderly to reduce functional disability.

In practice, the primary care physician’s role in the care of the elderly must move beyond the therapeutics of the single disease-oriented models to a holistic, patient-centred care. This paradigm shift, which functional status assessment promotes, must be emphasised in the training of medical students.

A well-coordinated care for these complicated and functionally impaired patients should be the goal of all health practitioners caring for the elderly. A short, but comprehensive functional assessment tool should be routinely used in consultations of the elderly to achieve this.

Further research to validate the various functional assessment tools to make them more culturally sensitive, thereby limiting the reporting bias, is needed. Studies assessing the IADL and AADL, which encompass a range of activities performed by a person living independently in a community, will also be useful. The impact of interventions on the functional status of an elderly population in this environment needs to be explored in prospective future studies.

The relevance of functional assessment with regard to prognostication and medical cost evaluation has not been explored in this environment. Studies along these lines would be of benefit to the clients, clinicians and policy makers.
